# Supplementary material for: Hydrogen bond unlocking-driven pore structure control for shifting multi-component gas separation function
Source: Nat Commun. 2024 Jan 27;15:804. doi: 10.1038/s41467-024-45081-w (PMC10821866; doi:10.1038/s41467-024-45081-w)
Supplement: Supplementary file 1 — Supplementary Information [file 41467_2024_45081_MOESM1_ESM.pdf]

Supplementary Information

Hydrogen Bond Unlocking-Driven Pore Structure Control for Shifting Multi-Component Gas Separation Function

Rong Yang,[1, †] Yu Wang,[1, †] Jian-Wei Cao,[1] Zi-Ming Ye,[2] Tony Pham,[3] Katherine A. Forrest,[3] Rajamani Krishna,[4] Hongwei Chen,[5] Libo Li,[5] Bo-Kai Ling,[1] Tao Zhang,[1] Tong Gao,[1] Xue Jiang,[1] Xiang-Ou Xu,[1] Qian-Hao Ye,[1] & Kai-Jie Chen[1,*]

[1]Key Laboratory of Special Functional and Smart Polymer Materials of Ministry of Industry and Information Technology, Xi'an Key Laboratory of Functional Organic Porous Materials, School of Chemistry and Chemical Engineering, Northwestern Polytechnical University, Xi'an, Shaanxi 710072, P.R. China

[2]Fujian Key Laboratory of Polymer Materials, College of Chemistry and Materials Science, Fujian Normal University, Fuzhou 350007, P.R. China

[3]Department of Chemistry, University of South Florida, 4202 East Fowler Avenue, CHE205, Tampa, Florida 33620-5250, USA

[4]Van 't Hoff Institute for Molecular Sciences, University of Amsterdam, Science Park 904, 1098 XH Amsterdam, The Netherlands.

[5]Shanxi Key Laboratory of Gas Energy Efficient and Clean Utilization, College of Chemical Engineering and Technology, Taiyuan University of Technology, Taiyuan, P.R. China.

*Corresponding authors: ckjiscon@nwpu.edu.cn

[†]R.Y. and Y.W. have contributed equally.

Table of Contents

Supplementary Methods S3
Supplementary Notes S4
Supplementary Figures S5
Supplementary Tables S22
Supplementary References S27

Supplementary Methods 1. Materials and Synthesis of Zn-fa-atz (2)/PES composite beads

Poly(ether sulfone) powder (PES, E6020, M_W~150000 by GPC, 99%) was purchased from BASF chemical company (Germany). Other materials: zinc nitrate hexahydrate ($Zn(NO_3)_2$•$6H_2O$, 99%), fumaric acid (H_2fa, 99%), 3-amino-1,2,4-triazole (Hatz, 96%), 1*H*-1,2,4-triazole-3,5-diamino (Hdatz, 98%), methanol (MeOH, 99%), N, N-dimethylformamide (DMF, 99%), were purchased and used without further purification.

He (99.999%), N_2 (99.999%), CO_2 (99.995%), C_2H_2 (99.9%), C_2H_4 (99.999%), and C_2H_6 (99.999%) were purchased from Jinan Heli Special Gas Co., Ltd (china).

The Zn-fa-atz (2)/PES beads were prepared via the phase inversion method: typically, 0.2 g of PES was dissolved in 1 mL of DMF. The mixture was stirred until PES was completely dissolved in DMF at room temperature. Then, 0.8 g of Zn-fa-atz (2) was dispersed in the above polymer solution and stirred for another 5 min. The resulting polymer slurry was vertically pipetted to a 100 mL MeOH coagulation bath (stirring on a magnetic stir plate) through a 1 mL fine stainless-steel syringe tip with a size of 21 gauge (0.8 mm in inner diameter). The air gap was about 6 cm, the droplets were slowly added to MeOH and immediately solidified to beads via solvent/MeOH exchange. The loading of Zn-fa-atz (2) in the obtained Zn-fa-atz (2)/PES composite beads is 80 wt% (considering there is no weight loss during the phase inversion method). Finally, the beads were transferred to 250 mL scintillation vial glass bottles filled with fresh MeOH and exchanged with fresh MeOH three times daily for three days.

Supplementary Methods 2. Physical Measurements

Powder X-ray diffraction (PXRD) patterns were recorded on a Rigaku Mini Flex II X-ray diffractometer (Cu K_α, λ = 1.54059 Å) with an operating power of 40 kV, 15 mA. All samples were subjected to scanning at 2θ from 5–50° with a scanning rate of 5 ^{o}/min. Thermogravimetric analyses (TGA) were conducted by using a METTLER TGA2 instrument in N_2 flow with a heating rate of 10 ^{o}C/min from 30 to 800 ^{o}C. Scanning Electron Microscopy (SEM) images were collected using the FEI Verios G4 SEM instrument at an acceleration voltage of 5–20 kV to investigate the surface and the cross-section morphology of Zn-fa-atz (2)/PES composite beads.

Supplementary Methods 3. Kinetic adsorption measurements

Time-dependent adsorption determination of $CO_2/C_2H_2/C_2H_4/C_2H_6$ was performed with a TA Instruments Discovery thermobalance at 298 K and 1 atm or an automatic volumetric adsorption apparatus BELSORP-HP at 298 K. Prior to each adsorption measurement, the samples were activated at 75 °C for 0.5/4 h under N_2 or a high vacuum. The resulting curves was used to calculate the diffusional time constants by using the following Eq.1 or Eq.2:

$$\frac{M_t}{M_e} = 1 - \exp(-kt) \qquad (1)$$

Where M_t is the mass uptake at time t, M_e is the mass uptake at equilibrium, and *k* is the kinetic rate constant.[1] After normalization of kinetic adsorption data, *t* (min) and M_t/M_e were taken as x and y axes, respectively.

$$\frac{M_t}{M_e} = \frac{6}{\sqrt{\pi}} \cdot \sqrt{\frac{D}{r^2}} \cdot \sqrt{t} \qquad (2)$$

Where M_t is the gas adsorbed amount at time t, M_e is the gas adsorbed amount at equilibrium, *D* is the diffusivity and *r* is the radius of the equivalent spherical particle.[2]Therefore, the slopes of q_t/q_∞ versus $\sqrt{t}$ are derived from the fitting of the plots in the low gas adsorbed amount range, and then *D*' is obtained from the square of the slope multiplying by π/36.

Supplementary Methods 4. Single X-ray diffraction characterization

Crystal data of Zn-fa-atz (2) were collected at 195 K and 298 K on a Bruker D8 Venture diffractometer equipped with Ga Kα microfocus X-ray generator (λ=1.34139 Å), Photon II detector. The data were empirically corrected for X-ray adsorption with Sadabs,[3,4] in the

Bruker APEX II software suite. The structures were solved by the direct method and refined with the full-matrix least-squares technique using the SHELXTL program package.[4,5] Anisotropic thermal parameters were applied to all non-hydrogen atoms. Hydrogen atoms were generated geometrically (C–H 0.95 Å). The Olex Solvent Mask treatment was applied because the guest molecule is extremely disordered and cannot be modeled. The crystallographic data of Zn-fa-atz (2) were summarized in Supplementary Table 3, and ORTEP style illustrations of the structure are provided in Supplementary Figure 13. The crystal structure has been deposited at the Cambridge Crystallographic Data Centre (CCDC), under deposition number CCDC 2176255-2176256, and can be obtained free of charge (http://www.ccdc.cam.ac.uk/data_request/cif).

Supplementary Notes 1. Calculation of adsorption enthalpy

$$\ln(P) = \ln(N) + \left(\frac{1}{T}\right)\sum_{i=0}^{m} a_i N^i + \sum_{j=0}^{n} b_j N^j \qquad (3)$$

In order to extract the coverage-dependent adsorption enthalpy, the isotherm data of Zn-fa-datz (1) and Zn-fa-atz (2) were fitted with a virial-type expression of the above form at 273 K and 298 K, where P is the pressure in kPa, N is the amount uptake in mmol/g, T is the temperature in K, a_i and b_j are virial coefficients that are independent of temperature, and m and n are the number of coefficients used to adequately describe the isotherm.

$$Q_{st} = -R\sum_{i=0}^{n} a_i N^i \qquad (4)$$

Where Q_{st} is the coverage-dependent enthalpy of adsorption in kJ mol^{-1}, R is the universal gas constant. The adsorption enthalpy at zero loading is calculated according to Supplementary Equation 4.

Supplementary Notes 2. Calculation of adsorption selectivity

The single-component gas isotherms for CO_2, C_2H_2, C_2H_4 and C_2H_6 in Zn-fa-datz (1) and Zn-fa-atz (2) at 298 K were fitted to the dual-site Langmuir-Freundlich equation:

$$q = Q_{A,sat}\frac{b_A P^{v_A}}{1 + b_A P^{v_A}} + Q_{B,sat}\frac{b_B P^{v_B}}{1 + b_B P^{v_B}} \qquad (5)$$

where $Q_{A,\,sat}$ and $Q_{B,\,sat}$ is the saturation uptakes (mmol/g) for sites A and B, P is the pressure in kPa, b_A and b_B are the affinity coefficients (in kPa^{-vi}) of sites A and B.

The adsorption selectivity of adsorbate i relative to adsorbate j was established by using the Ideal Adsorption Solution Theory (IAST).[6] The equation is shown in the following:

$$S_{i/j} = \frac{x_i}{x_j}\frac{y_j}{y_i} \qquad (6)$$

here, x_i and x_j are the equilibrium adsorption capacity of components i and j, respectively, in the adsorbed phase, and y_i and y_j are the mole fractions of components i and j, in the gas phase.

Supplementary Methods 5. Transient breakthrough experiments vs simulations for Zn-fa-atz (2)

Transient breakthrough experiments were carried out for ternary $C_2H_2/C_2H_4/C_2H_6$ and quaternary $CO_2/C_2H_2/C_2H_4/C_2H_6$ mixtures at a total pressure of 100 kPa and 298 K. Corresponding desorption experiments were also conducted using Helium as purge gas.

Transient breakthrough simulations were carried out for the same set of operating conditions as in the experimental data sets, using the methodology described in earlier publications. [7-11]In these simulations, intra-crystalline diffusion influences are ignored.

Supplementary Methods 6. Pawley and Rietveld refinement of PXRD

The microcrystalline Zn-fa-datz (1) and Zn-fa-atz (2) was placed in a glass capillary (Φ = 0.8 mm) connected with an automatic volumetric sorption apparatus (Micromertics 3FLEX), and heated under high vacuum at 75 °C for 4 hours. After that, CO_2, C_2H_2, C_2H_4 and C_2H_6 gas was introduced by cooling the samples with dry ice-acetone bath to 195K, and the gas dosed volumetrically from calibrated pressure. PXRD data of the gas-loaded samples was collected on a Rigaku SmartLab X-ray powder diffractometer (Cu Kα) with a scanning speed of 0.01 ^{o}/step and 7 s/step under capillary transmission mode. All the indexing and refinement were performed by the Reflex plus module of Material Studio 5.0. The pseudo-Voigt profile parameters, background parameters, the cell parameters, the zero point of the diffraction pattern, the global isotropic atom displacement parameters, the Berar-Baldinozzi asymmetry correction parameters, and the March-Dollase preferred orientation correction parameters were optimized step by step to improve the agreement between the calculated and the experimental powder diffraction patterns.

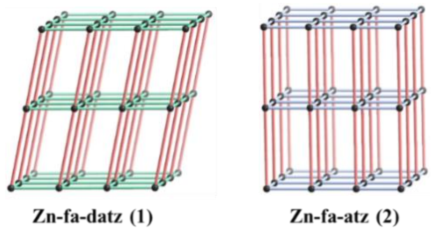

Supplementary Figure 1. Topology of Zn-fa-datz (1) (left) and Zn-fa-atz (2) (right) (dinuclear Zn, triazolate, and dicarboxylate are simplified as black sphere, green or blue stick, and red stick, respectively).

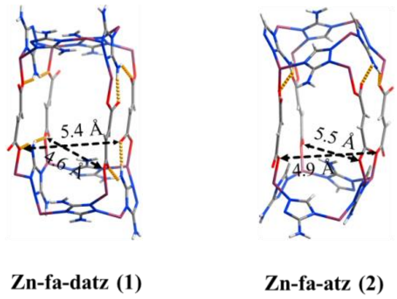

Supplementary Figure 2. The pore size of cage in Zn-fa-datz (1) (left) and Zn-fa-atz (2) (right).

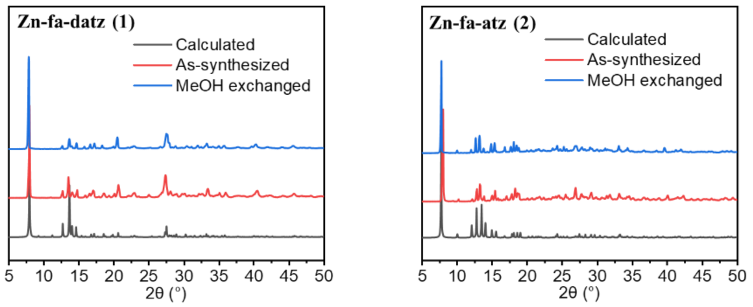

Supplementary Figure 3. PXRD patterns of Zn-fa-datz (1) (left) and Zn-fa-atz (2) (right).

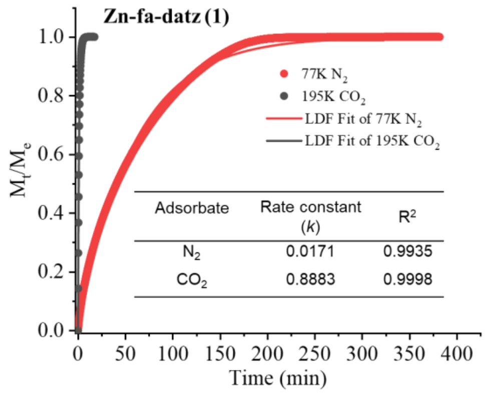

Supplementary Figure 4. Diffusional rate constants of N_2 (77 K) and CO_2 (195 K) in Zn-fa-datz (1).

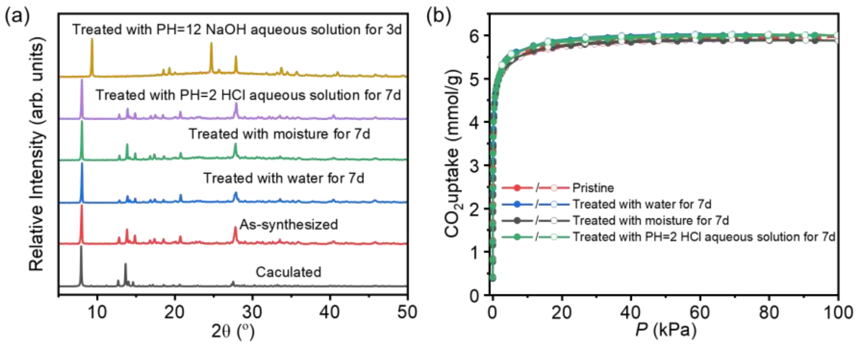

Supplementary Figure 5. PXRD patterns (a) and CO_2 adsorption/desorption isotherms (b) of Zn-fa-datz (1) at 195 K after different treatments. After being exposed to water or moisture (*ca.* 35% RH), or acidic aqueous solutions (pH=2) for 7 days, the PXRD and CO_2 uptakes of the treated samples remain almost unchanged, while obvious change was observed in pH=12 after 3 days, demonstrating the exceptional acid stability but poor alkaline stability of Zn-fa-datz (1).

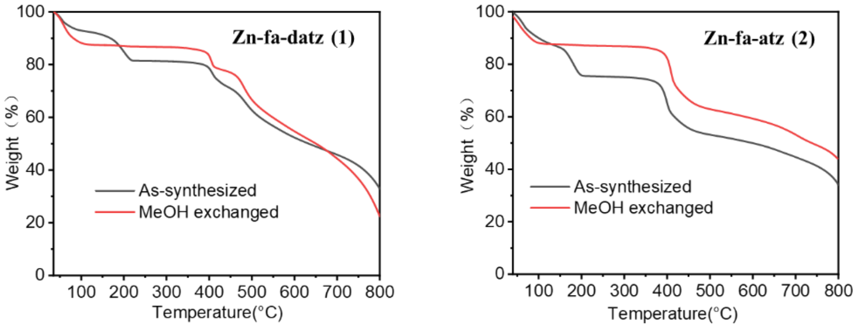

Supplementary Figure 6. TGA curves of Zn-fa-datz (1) (left) and Zn-fa-atz (2) (right).

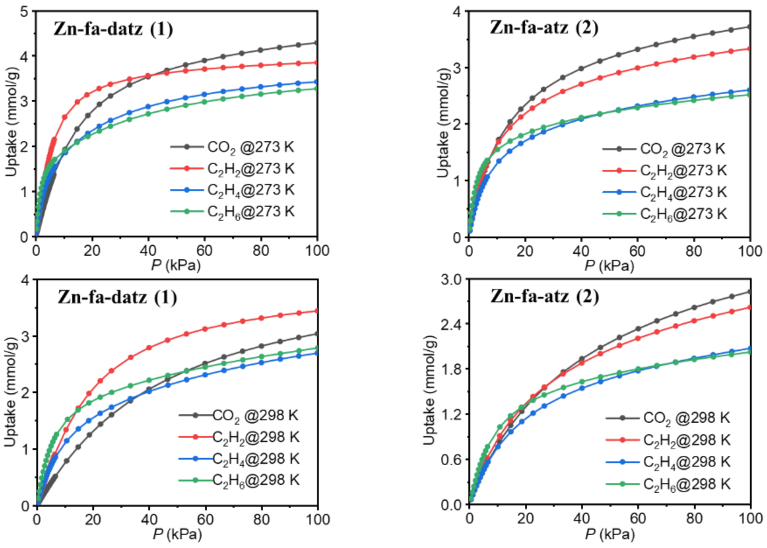

Supplementary Figure 7. Adsorption isotherms of Zn-fa-datz (1) (left) and Zn-fa-atz (2) (right) for CO_2 (black), C_2H_2 (red), C_2H_4 (blue), and C_2H_6 (green) at 273 K and 298 K, respectively.

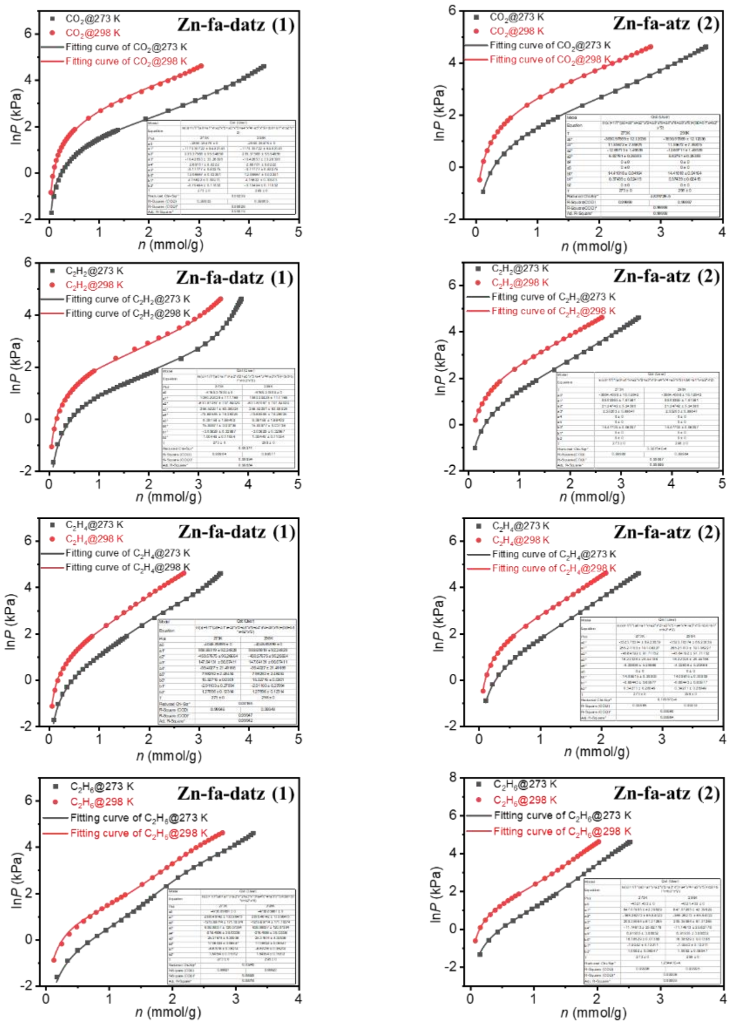

Supplementary Figure 8. Virial fittings of CO_2 , C_2H_2, C_2H_4, and C_2H_6 adsorption isotherms of Zn-fa-datz (1) (left) and Zn-fa-atz (2) (right).

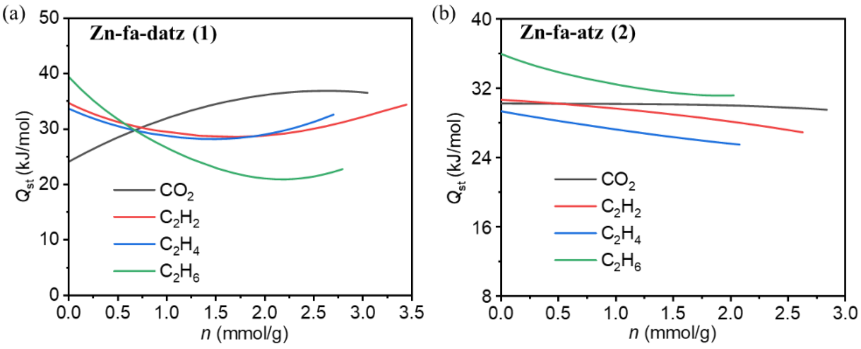

Supplementary Figure 9. Gas adsorption enthalpies of Zn-fa-datz (1) (a), Zn-fa-atz (2) (b) calculated by virial method.

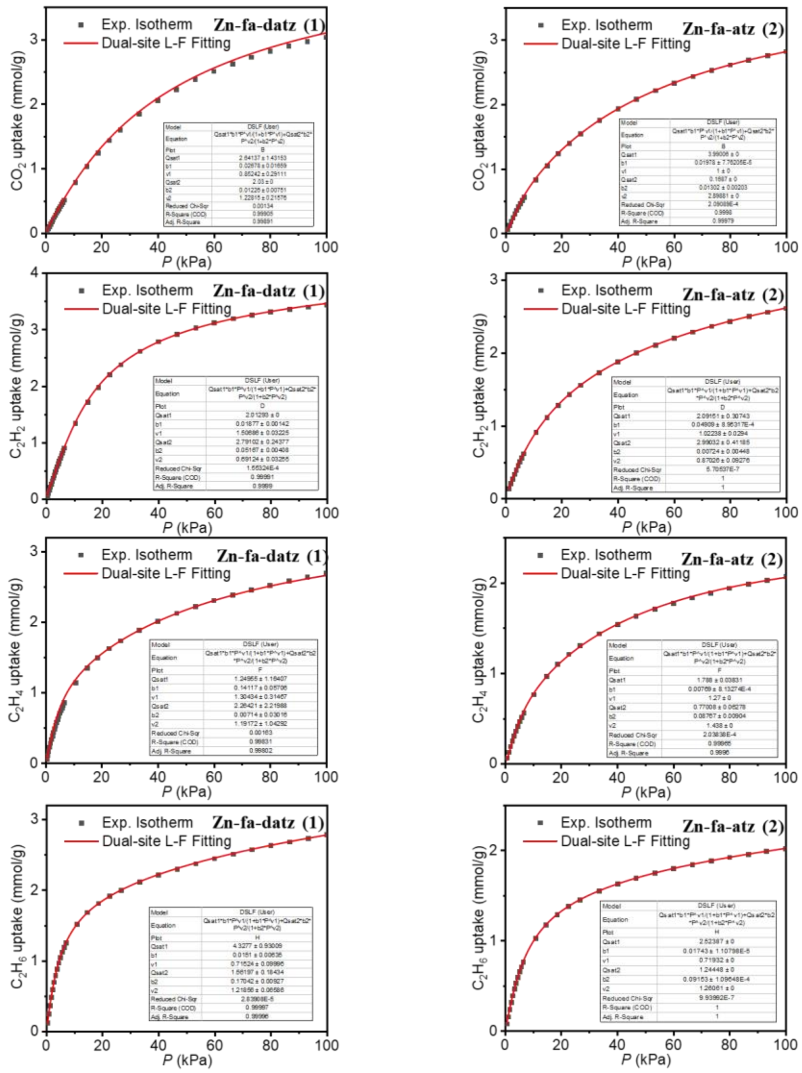

Supplementary Figure 10. Dual-site Langmuir-Freundlich fittings of CO_2, C_2H_2, C_2H_4, and C_2H_6 adsorption isotherms of Zn-fa-datz (1) (left) and Zn-fa-atz (2) (right).

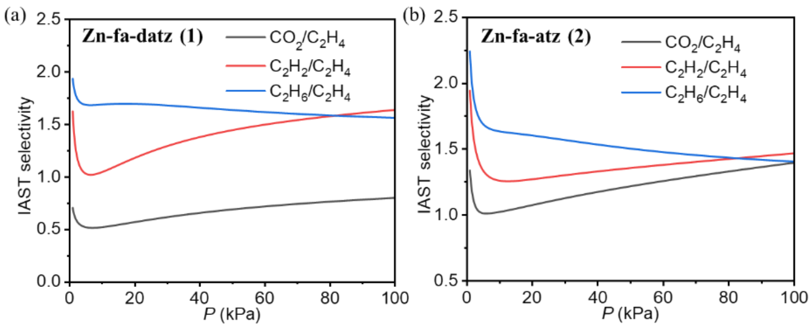

Supplementary Figure 11. Ideal Adsorbed Solution Theory (IAST) selectivities of Zn-fa-datz (1) (a), Zn-fa-atz (2) (b) using the dual-site Langmuir-Freundlich model .

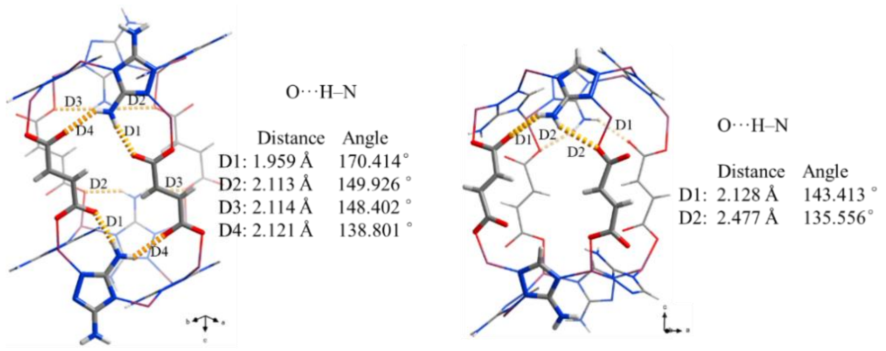

Zn-fa-datz (1) **Zn-fa-atz (2)**

Supplementary Figure 12. The hydrogen bond parameters of Zn-fa-datz (1) and Zn-fa-atz (2).

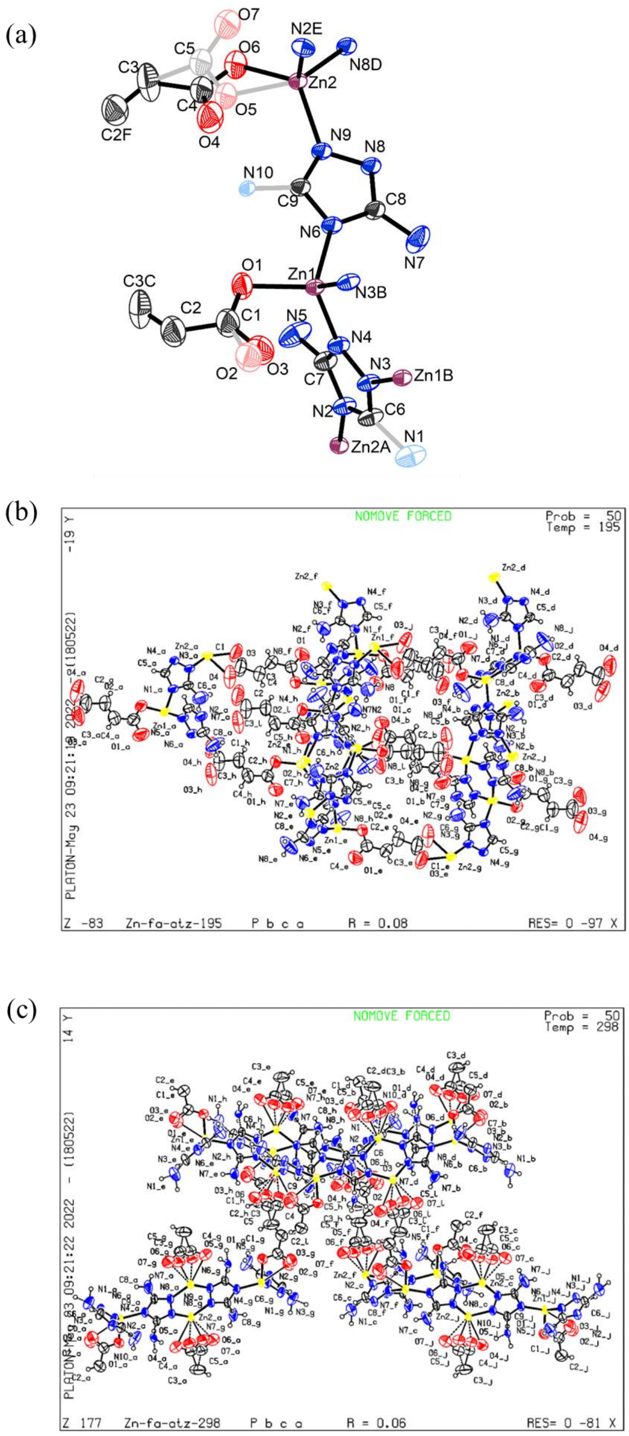

Supplementary Figure 13. Coordination environment of Zn-fa-atz (2). (a) Thermal ellipsoids (30% probability) drawn by Diamond. Hydrogen atoms are omitted for clarity, symmetric codes: A, 1/2-x, -1/2+y, z; B, -x, 1-y, 1-z; C, -1/2+x, y, 1/2-z; D, 1-x, 1-y, 1-z; E, 1/2-x, 1/2+y, z; F, 1/2+x, y, 1/2-z, ORTEP drawing of the Zn-fa-atz (2) of (b) 195 K and (c) 298K, produced by the checkCIF report of the International Union of Crystallography. CCDC deposit number 2176255 and 2176256, respectively.

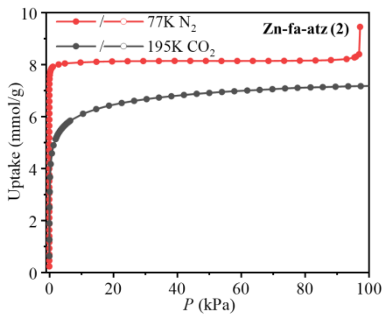

Supplementary Figure 14. Comparison of the adsorption isotherms of 77 K N_2 (red) and 195 K CO_2 (black) for Zn-fa-atz (2).

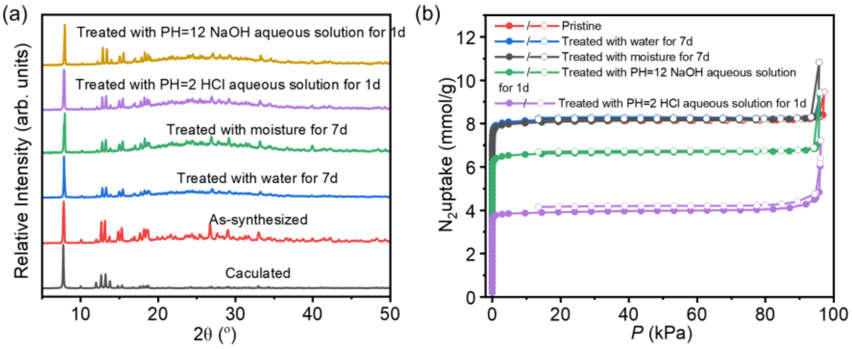

Supplementary Figure 15. PXRD patterns (a) and N_2 adsorption/desorption isotherms (b) of Zn-fa-atz (2) at 77 K after different treatments. After being exposed to water or moisture conditions (*ca.* 35% RH) for 7 days, the PXRD peak intensities and N_2 uptakes of the treated Zn-fa-atz (2) samples remain the same, indicating the exceptional chemical stability of Zn-fa-atz (2) under aqueous and moisture conditions. However, when Zn-fa-atz (2) was exposed to acid (pH=2) or alkaline aqueous (pH=12), the N_2 uptakes of the treated samples dramatically changed after 1 day but the PXRD remain almost unchanged.

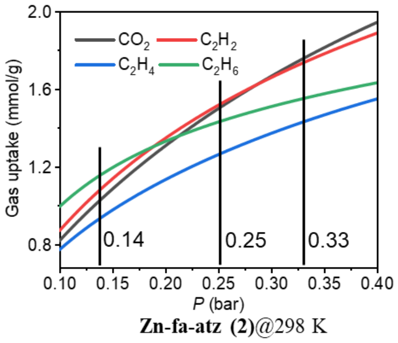

Supplementary Figure 16. Single-component adsorption isotherms of Zn-fa-atz (2) for CO_2 (black), C_2H_2 (red), C_2H_4 (blue) and C_2H_6 (green) from 0.1–0.4 bar and at 298 K obtained from dual-site Langmuir-Freundlich fittings.

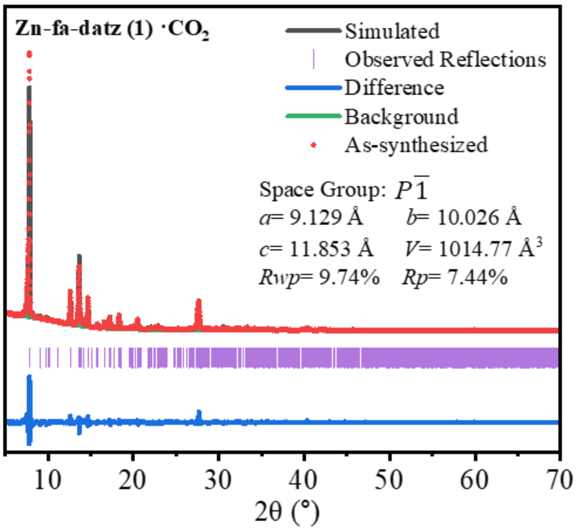

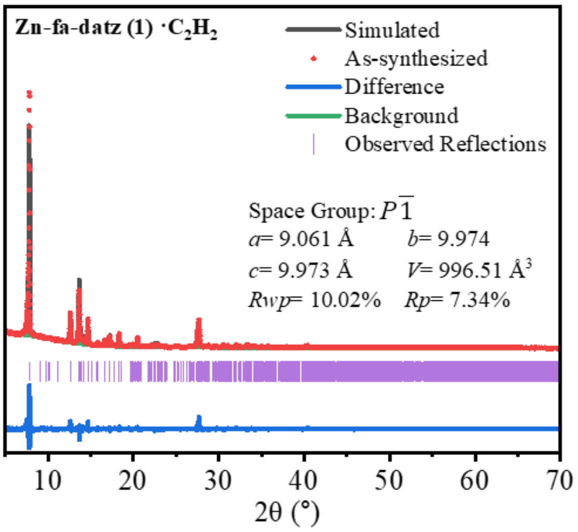

Supplementary Figure 17. Rietveld refinement plots of powder X-ray diffraction data of CO_2-loaded Zn-fa-datz (1) and C_2H_2-loaded Zn-fa-datz (1).

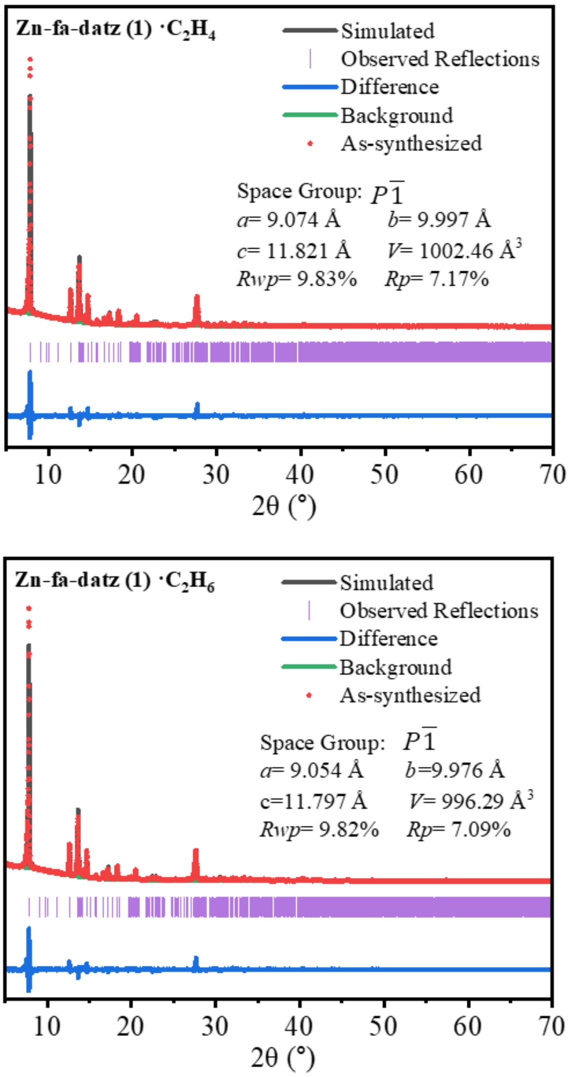

Supplementary Figure 18. Rietveld refinement plots of powder X-ray diffraction data of C_2H_4-loaded Zn-fa-datz (1) and C_2H_6-loaded Zn-fa-datz (1).

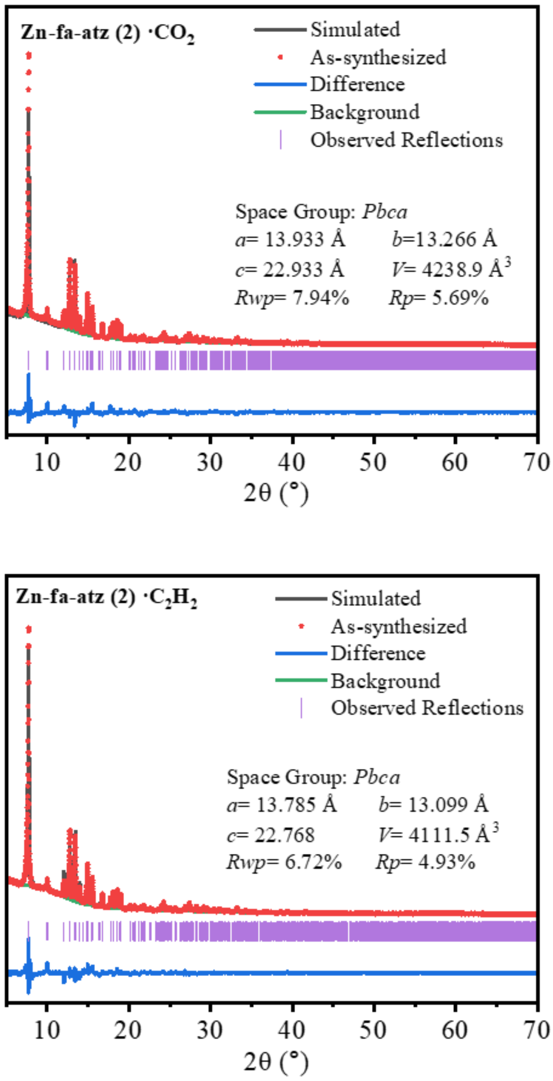

Supplementary Figure 19. Rietveld refinement plots of powder X-ray diffraction data of CO_2-loaded Zn-fa-atz (2) and C_2H_2-loaded Zn-fa-atz (2).

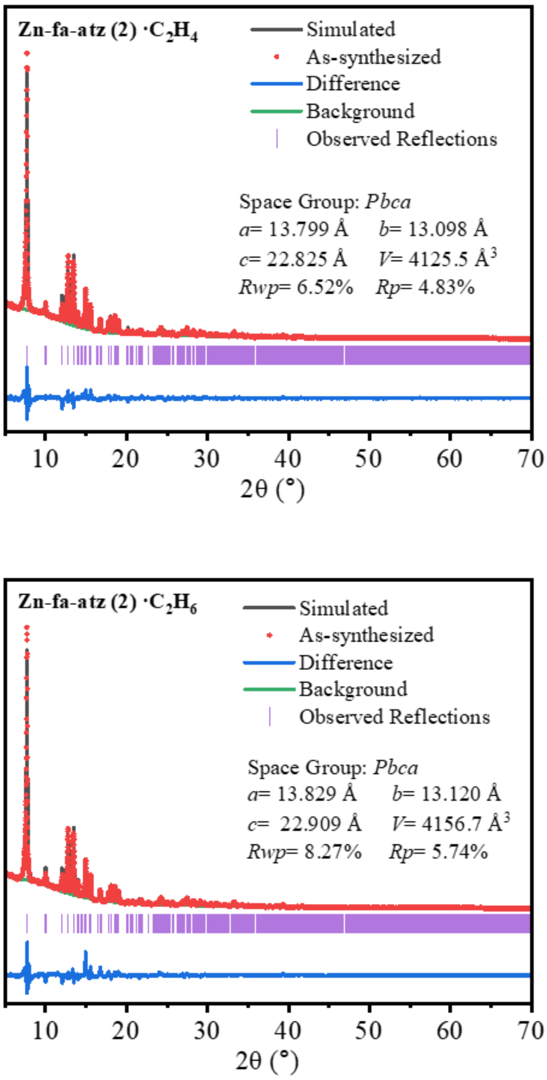

Supplementary Figure 20. Rietveld refinement plots of powder X-ray diffraction data of C_2H_4-loaded Zn-fa-atz (2) and C_2H_6-loaded Zn-fa-atz (2).

Retention time	Components name	Peak area
136.38	C_2H_4	1615.61

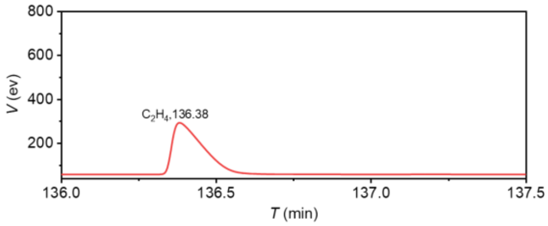

Retention time	Components name	Peak area
139.35	C_2H_4	2961.96
139.73	C_2H_6	1.94
140.20	C_2H_2	9.03

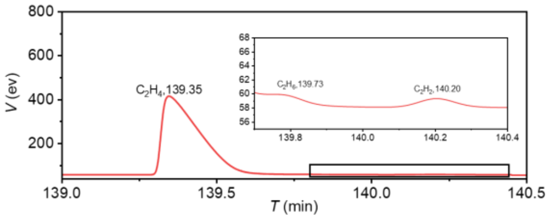

Supplementary Figure 21. GC profiles for C_2H_4 at the outlet of Zn-fa-atz (2) column in a $CO_2/C_2H_2/C_2H_4/C_2H_6$ (1:1:1:1) mixture with a total gas flow of 2 mL/min.

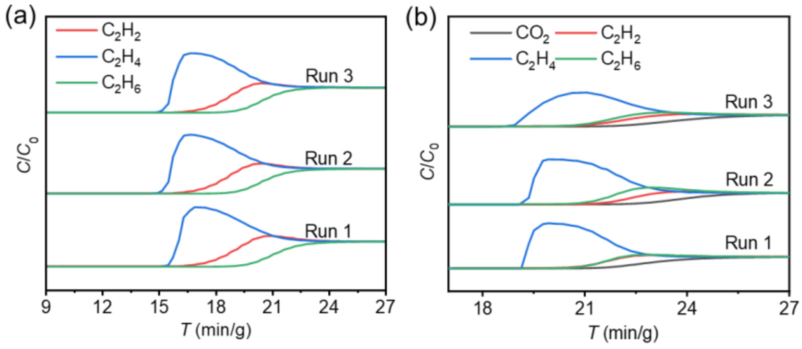

Supplementary Figure 22. Cycling column breakthrough tests of $C_2H_2/C_2H_4/C_2H_6$/He (a), and equimolar $CO_2/C_2H_2/C_2H_4/C_2H_6$ (b) mixture by using Zn-fa-atz (2) at room temperature and 1 atm.

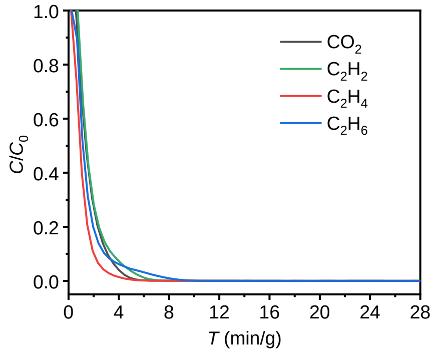

Supplementary Figure 23. Desorption curves of Zn-fa-atz (2) at 70 ºC in He flow with a rate of 20 mL/min.

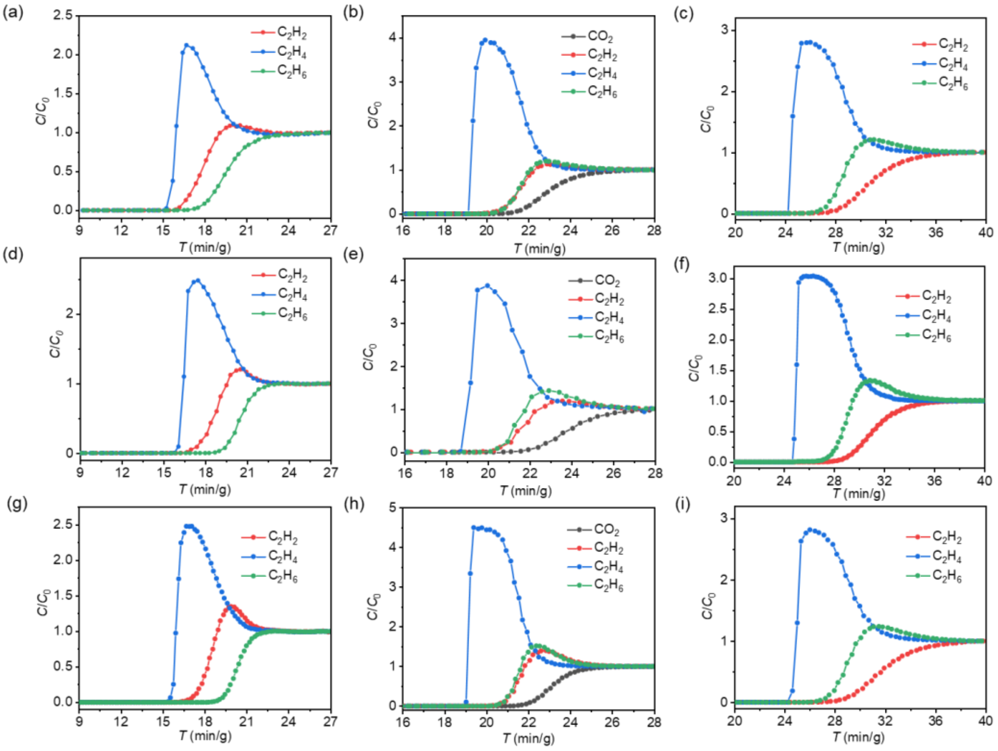

Supplementary Figure 24. Breakthrough curves of Zn-fa-atz (2) with different filling capacities, (a, b, c) the initial batch, (d, e, f) the second batch, (g, h, i) the third batch, using (a, d, g) $C_2H_2/C_2H_4/C_2H_6$/He mixture (1:1:1:4, *v*/*v*/*v*/*v*), (b, e, h) equimolar $CO_2/C_2H_2/C_2H_4/C_2H_6$ mixture (1:1:1:1, *v*/*v*/*v*/*v*) and (c, f, i) $C_2H_2/C_2H_4/C_2H_6$ mixture (1:1:1, *v*/*v*/*v*) at room temperature and 1 atm, respectively.

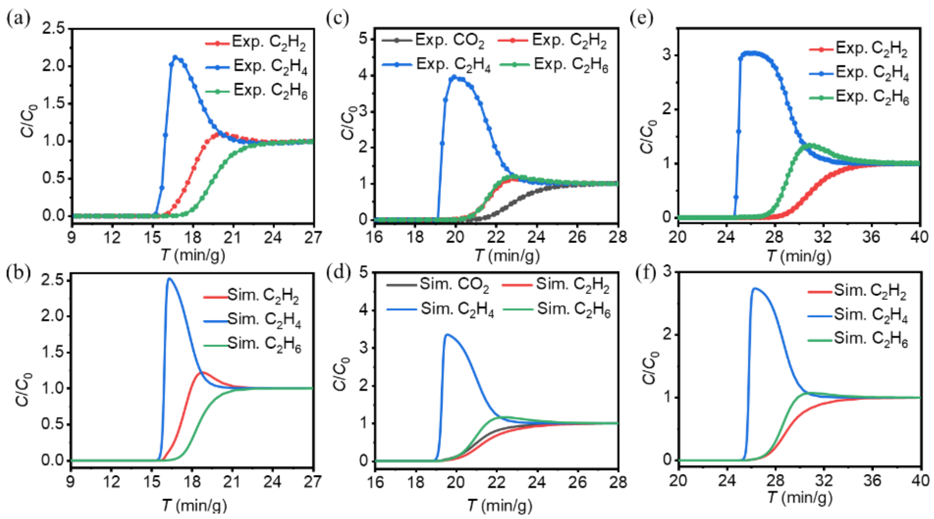

Supplementary Figure 25. Experimental and simulated column breakthrough curves at 298 K of Zn-fa-atz (2) powder for (a, b) $C_2H_2/C_2H_4/C_2H_6$/He, (c, d) $CO_2/C_2H_2/C_2H_4/C_2H_6$ and (e, f) $C_2H_2/C_2H_4/C_2H_6$ separations.

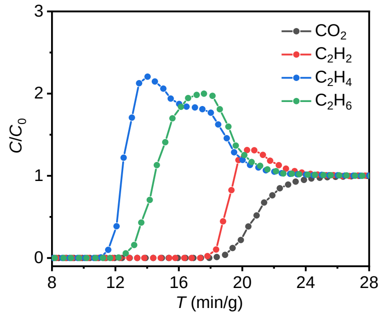

Supplementary Figure 26. Experimental breakthrough curves for quaternary $CO_2/C_2H_2/C_2H_4/C_2H_6$ mixtures (1:1:1:1, *v*/*v*/*v*/*v*) on Zn-fa-atz (2) under wet (*ca.* 36% RH) conditions at room temperature and 1 bar.

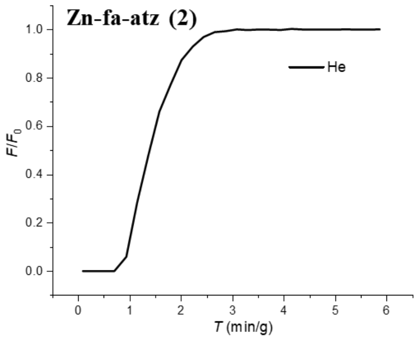

Supplementary Figure 27. Experimental breakthrough curves for He of 2 mL/min in Zn-fa-atz (2) at room temperature and 1 bar.

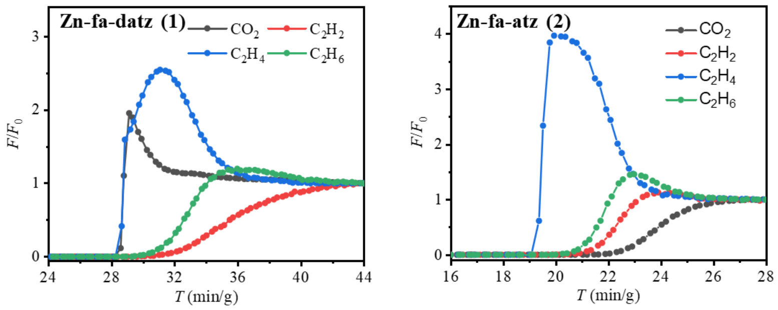

Supplementary Figure 28. Experimental breakthrough curves of Zn-fa-datz (1) and Zn-fa-atz (2) for quaternary $CO_2/C_2H_2/C_2H_4/C_2H_6$ mixtures (1:1:1:1, *v*/*v*/*v*/*v*) with velocity correction.

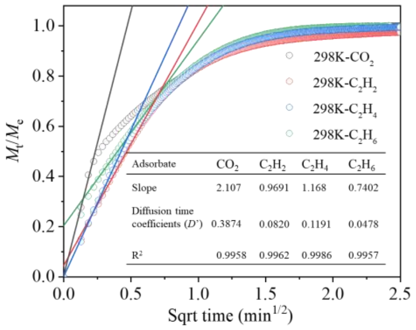

Supplementary Figure 29. Adsorption kinetics profiles (point) and linear fittings (line) of CO_2 (black), C_2H_2 (red), C_2H_4 (blue) and C_2H_6 (green) for Zn-fa-atz (2) at 298 K.

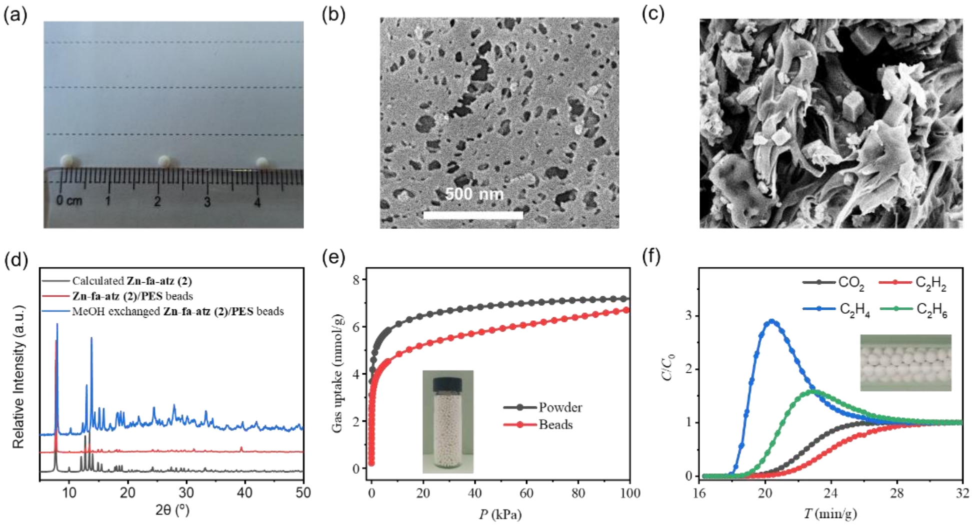

Supplementary Figure 30. (a) Physical appearance image of Zn-fa-atz (2)/PES beads. (b) SEM images of Zn-fa-atz (2)/PES bead surface and (c) the cross-section. (d) PXRD patterns of Zn-fa-atz (2)/PES beads. (e) Comparison of 195 K CO_2 adsorption isotherms of Zn-fa-atz (2) powder and Zn-fa-atz (2)/PES beads. (f) Experimental column breakthrough curves for Zn-fa-atz (2)/PES beads using an equimolar mixture of $CO_2/C_2H_2/C_2H_4/C_2H_6$ under same condition.

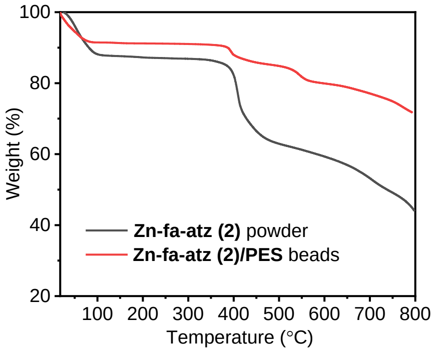

Supplementary Figure 31. Comparison of TGA curves of Zn-fa-atz (2) powder and Zn-fa-atz (2)/PES beads after MeOH exchanged.

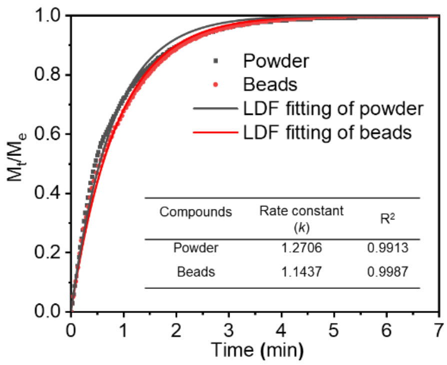

Supplementary Figure 32. Details of diffusional rate constants calculation for Zn-fa-atz (2) powder and Zn-fa-atz (2)/PES beads.

Supplementary Table 1. Summary of the structural information, adsorption data of **Zn-fa-datz (1) and Zn-fa-atz (2)**.

Compound	Zn-fa-atz (2)			Zn-fa-datz (1)	
Temperature	298 K	195 K	77 K	293 K	195 K
Topology	**pcu**			**pcu**	
Pore size[a]		6.3			6.1
Density (g cm^{-3})	1.284	1.336		1.428	
Volume ratio[b]	46.3	42.6		38.9	
Pore volume (measured)[c]	0.361	0.319		0.272	
Pore volume (measured from the uptake of the CO_2 isotherm)[d]		0.285			0.237
Pore volume (measured from the uptake of the N_2 isotherm)[e]			0.283		

[a] Pore size (Å) calculated based on the CO_2 isotherm at 195 K according to the Horvath-Kawazoe model (pore geometry: cylinder). [b] Volume ratio (%) estimated by PLATON software[12] without consideration of the solvent in the pore. [c] The calculated pore volume (cm^3 g^{-1}) estimated from single crystal data using PLATON software. [d] The measured pore volume (cm^3 g^{-1}) calculated from the uptake at P/P_0 = 0.96 assuming the liquid CO_2 filling in the pore. [e] The measured pore volume (cm^3 g^{-1}) calculated from the uptake of the N_2 isotherm at 77 K assuming the liquid N_2 filling in the pore, at P/P_0 = 0.95 for Zn-fa-atz (2).

Supplementary Table 2. Summary of the selectivity and gas adsorption enthalpy data of Zn-fa-datz (1) and Zn-fa-atz (2).

Compounds	Q_{st}[a]				IAST selectivity[b]		
	CO_2	C_2H_2	C_2H_4	C_2H_6	CO_2/C_2H_4	C_2H_2/C_2H_4	C_2H_6/C_2H_4
Zn-fa-atz (2)	30.2	30.6	29.3	35.9	1.4	1.5	1.4
Zn-fa-datz (1)	24.0	34.7	33.6	39.4	0.8	1.6	1.6

[a] Gas adsorption enthalpy (Q_{st}, kJ mol^{-1}). [b] Calculated from IAST theory with a ratio of 50:50 at 298 K and 100 kPa.

Supplementary Table 3. Summary of the single-crystal diffraction data.

Compounds	Zn-fa-atz (2)	Zn-fa-atz (2)	Zn-fa-datz (1)[c]
Empirical formula	$C_8H_8N_8O_4Zn_2$	$C_8H_8N_8O_4Zn_2$	$C_8H_{10}N_{10}O_4Zn_2$
Formula weight	410.96	410.96	441.04
Temperature (K)	298(2)	195(2)	293
Crystal system	orthorhombic	orthorhombic	triclinic
Space group	*Pbca*	*Pbca*	$P\overline{1}$
a (Å)	13.978(8)	13.9330(8)	9.1754(7)
b (Å)	13.376(13)	12.7549(7)	10.0864(7)
c (Å)	22.750(30)	22.9989(12)	11.9515(8)
α (°)	90	90	107.567(6)
β (°)	90	90	101.469(6)
γ (°)	90	90	92.975(6)
V (Å^3)	4253(33)	4087.2(4)	1025.99(13)
Z	8	8	2
D_c (g cm^{-3})	1.284	1.336	1.428
Reflns coll.	4191	3613	
R_{int}	0.0630	0.0796	
R_1 [$I > 2\sigma(I)$][a]	0.0586	0.0792	
wR_2 [$I > 2\sigma(I)$][b]	0.1706	0.2552	
R_1 (all data)	0.0752	0.1020	
wR_2 (all data)	0.1851	0.2728	
GOF	1.057	1.069	

[a] $R_1 = \Sigma||F_o| - |F_c||/\Sigma|F_o|$. [b] $wR_2 = [\Sigma w(F_o^2 - F_c^2)^2/\Sigma w(F_o^2)^2]^{1/2}$. [c] Adopted from ref[13].

Supplementary Table 4. Comparison of C_2H_6/C_2H_4, C_2H_2/C_2H_4 and CO_2/C_2H_4 selectivity in porous materials for one-step C_2H_4 purification for three-component, four-component mixtures at 298 K.

Gas mixtures	Adsorbent	IAST selectivity			References
		C_2H_6/C_2H_4	C_2H_2/C_2H_4	CO_2/C_2H_4	
$C_2H_2/C_2H_4/C_2H_6$	**[Zn(BDC)(H$_2$BPZ)]**	2.2[b]	1.6[b]	-	[14]
	**Zn(ad)(int)**	2.4[b]	1.61[a]	-	[15]
	**TJT-100**	1.2[a]	1.8[a]	-	[16]
	**Azole-Th-1**	1.46[b]	1.09[b]	-	[17]
	**NPU-1**	1.32[b]	1.4[b]	-	[18]
	**UPC-612**	1.4[b]	1.07[b]	-	[19]
	**UPC-613**	1.5[b]	1.4[b]	-	
	**UIO-67-(NH$_2$)$_2$**	1.7[b]	2.1[a]	-	[20]
	**Ni-BDC-INA**	1.56[b]	1.37[b]	-	[21]
	**CPM-173**	1.76[b]	1.51[b]	-	
	**UiO-66**	1.57[b]	1.45[b]	-	
	**DMOF-1**	1.51[b]	1.35[b]	-	
	**CuTiF6-TPPY**	2.12[b]	5.47[b]	-	[22]
	**ZJNU-115**	1.56[b]	2.05[a]	-	[23]
	**ZJNU-7**	1.56[b]	1.77[a]	-	[24]
	**NUM-9a**	1.62[b]	1.48[a]	-	[25]
	**MIL-125**	1.21[b]	2.32[b]	-	[26]
	**NH$_2$-MIL-125**	1.18[b]	3.75[b]	-	
	**ZSTU-2**	1.62[b]	2.36[b]	-	
	**LIFM-XYY-6**	1.63[b]	1.53[a]	-	[27]
	**UPC-66-a**	1.65[b]	1.05[b]	-	[28]
	**Zn-ATA**	1.84[a]	1.81[a]	-	[29]
	**HIAM-210**	2.0[b]	2.0[b]	-	[30]
	**HIAM-326**	1.9[b]	1.6[b]	-	[31]
	**Zn-fa-datz (1)**	1.4[b]	1.5[b]	0.8[b]	**This work**
$C_2H_2/C_2H_4/C_2H_6/CO_2$	**Zn-atz-oba**	1.27[b]	1.43[b]	1.33[b]	[32]
	**Al-MOFM$_{15}$[c]**	2.51[b]	3.32[b]	-	[33]
	**Zn-fa-atz (2)**	1.6[b]	1.6[b]	1.4[b]	**This work**

[a] IAST selectivity for 1/99 gas mixture. [b] IAST selectivity for 1/1 gas mixture. [c] IAST selectivity calculated at 293K.

Supplementary Table 5. Host-guest interactions in PXRD analyses and structural refinements of Zn-fa-datz (1) and Zn-fa-atz (2) loaded with CO_2, C_2H_2, C_2H_4 and C_2H_6.

	Zn-fa-datz (1)				Zn-fa-atz (2)			
Guest molecules		H···A (Å)	D-H···A (º)	D···A (Å)		H···A (Å)	D-H···A (º)	D···A (Å)
C_2H_2	C51-H53···N48	2.818	163.268	3.856	C24-H26···N94	3.852	121.854	4.499
	C51-H53···N7	3.825	132.105	4.612				
C_2H_4	C50-H53···N44	1.829	164.048	2.895	C93-H96···N125	3.022	126.05	3.747
	C50-H53···N43	2.251	131.006	3.080	C93-H96···O45	3.277	160.87	4.295
	C50-H53···N45	2.564	134.221	3.416	C92-H95···O3	3.507	121.13	4.157
	C50-H52···N21	2.651	163.689	3.712	C92-H94···O46	3.545	149.50	4.516
	C50-H53···N67	2.682	126.992	3.451	C92-H95···N101	3.584	168.60	4.630
	C50-H53···N48	3.148	127.414	3.909	C93-H97···O67	3.987	124.60	4.691
C_2H_6	C77-H81···N52	1.960	130.758	2.805	C34-H38···N4	1.971	167.94	3.065
	C77-H81···N51	1.998	160.316	3.057	C35-H41···O44	2.137	136.29	3.037
	C77-H79···N28	2.236	149.066	3.230	C34-H36···O15	2.888	148.93	3.883
	C77-H79···N5	2.388	156.829	3.428	C35-H40···N61	3.471	123.23	4.183
	C77-H81···N53	2.395	142.087	3.333	C34-H38···O74	3.938	127.02	4.690
	C78-H84···O65	2.666	144.85	3.622				
	C77-H80···N21	2.751	131.255	3.574				
	C77-H79···N29	2.848	155.325	3.876				
	C77-H80···N60	2.911	141.129	3.831				
	C77-H79···N32	3.329	128.931	4.111				
	C77-H79···N7	3.552	141.687	4.468				
	C78-H83···O16	3.930	142.316	4.848				
CO_2	C98···O1			3.641	C34···O71			3.231
	C98···O2			3.773	C34···O108			3.883
	C98···N7			3.810	O36···C103			3.337
	C98···O3			3.837	O35···C72			3.800
	C98···N14			3.886	O35···C73			3.805
	O99···C3			3.357	O35···C115			3.808
	O99···C6			3.603	O36···C72			3.864
	O99···C5			3.685				
	O99···C4			3.801				
	O97···C3			3.970				

Supplementary Table 6. The adsorption uptake from single-component isotherms and breakthrough time sequence of various gases under different breakthrough conditions for Zn-fa-atz (2).

Gas flow rate (mL/min)		$C_2H_2/C_2H_4/C_2H_6$/He 0.5/0.5/0.5/2	$C_2H_2/C_2H_4/C_2H_6$ 0.5/0.5/0.5	$CO_2/C_2H_2/C_2H_4/C_2H_6$ 0.5/0.5/0.5/0.5
Gas uptake at 298 K (mmol/g)	C_2H_2	1.1[a]	1.7[b]	1.5[c]
	C_2H_4	0.94[a]	1.4[b]	1.3[c]
	C_2H_6	1.2[a]	1.6[b]	1.4[c]
	CO_2			1.5[c]
	uptake sequence	$C_2H_4<C_2H_2<C_2H_6$	$C_2H_4<C_2H_6<C_2H_2$	$C_2H_4<C_2H_6<C_2H_2\approx CO_2$
Breakthrough time sequence	initial	$C_2H_4<C_2H_2<C_2H_6$	$C_2H_4<C_2H_6<C_2H_2$	$C_2H_4<C_2H_6<C_2H_2\approx CO_2$
	2nd	$C_2H_4<C_2H_2<C_2H_6$	$C_2H_4<C_2H_6<C_2H_2$	$C_2H_4<C_2H_6<C_2H_2\approx CO_2$
	3rd	$C_2H_4<C_2H_2<C_2H_6$	$C_2H_4<C_2H_6<C_2H_2$	$C_2H_4<C_2H_6<C_2H_2\approx CO_2$

[a] The single-component gas adsorption uptake data calculated by dual-site Langmuir-Freundlich fittings at partial pressure of 0.14 bar for C2 gases. [b]. The gas uptake data at partial pressure of 0.33 bar. [c] The gas uptake data at partial pressure of 0.25 bar.

Supplementary Table 7. The $CO_2/C_2H_2/C_2H_4/C_2H_6$ breakthrough performances of Zn-fa-datz (1).

Zn-fa-datz (1)	CO_2 uptake (mmol/g)	C_2H_2 uptake (mmol/g)	C_2H_4 uptake (mmol/g)	C_2H_6 uptake (mmol/g)	*S* (C_2H_6/C_2H_4)	*S* (C_2H_2/C_2H_4)	*S* (CO_2/C_2H_4)
Adsorption isotherm at 0.25 bar (298 K)[a]	1.54	2.31	1.69	1.97	-	-	-
Breakthrough experiment	0.52	0.73	0.41	0.63	1.6	1.6	0.8
IAST	-	-	-	-	1.54	1.78	1.27

[a] The single-component gas adsorption uptake data calculated by dual-site Langmuir-Freundlich fittings at partial pressure of 0.25 bar for C2 gases.

Supplementary Table 8. The $CO_2/C_2H_2/C_2H_4/C_2H_6$ breakthrough performances of Zn-fa-atz (2).

**Zn-fa-atz (2)**	CO_2 uptake (mmol/g)	C_2H_2 uptake (mmol/g)	C_2H_4 uptake (mmol/g)	C_2H_6 uptake (mmol/g)	*S* (C_2H_6/C_2H_4)	*S* (C_2H_2/C_2H_4)	*S* (CO_2/C_2H_4)
Adsorption isotherm at 0.25 bar (298 K)	1.5	1.5	1.3	1.4	-	-	-
Breakthrough experiment at 1 bar (298 K)	0.50	0.46	0.23	0.43	1.87	2.00	2.17
IAST	-	-	-	-	1.4	1.5	1.4

Supplementary References

1. Reid, C. R. & Thomas, K. M. Adsorption of Gases on a Carbon Molecular Sieve Used for Air Separation: Linear Adsorptives as Probes for Kinetic Selectivity. *Langmuir* **15**, 3206-3218 (1999).
2. Lee, C. Y. *et al.* Kinetic separation of propene and propane in metal−organic frameworks: controlling diffusion rates in plate-shaped crystals via tuning of pore apertures and crystallite aspect ratios. *J. Am. Chem. Soc.* **133**, 5228-5231 (2011).
3. Dolomanov, O. V., Bourhis, L. J., Gildea, R. J., Howard, J. A. & Puschmann, H. OLEX2: a complete structure solution, refinement and analysis program. *J. Appl. Crystallogr.* **42**, 339-341 (2009).
4. Sheldrick, G. M. A short history of SHELX. *Acta Crystallogr. A.* **64**, 112-122 (2008).
5. Sheldrick, G. M. Crystal structure refinement with SHELXL. *Acta Crystallogr. C.* **71**, 3-8 (2015).
6. Myers, A. L. & Prausnitz, J. M. Thermodynamics of mixed-gas adsorption. *AIChE J.* **11**, 121-127 (1965).
7. Krishna, R. Metrics for Evaluation and Screening of Metal-Organic Frameworks for Applications in Mixture Separations. *ACS omega* **5**, 16987-17004 (2020).
8. Krishna, R. The Maxwell–Stefan description of mixture diffusion in nanoporous crystalline materials. *Micropor. Mesopor. Mat.* **185**, 30-50 (2014).
9. Krishna, R. Methodologies for evaluation of metal–organic frameworks in separation applications. *RSC Adv.* **5**, 52269-52295 (2015).
10. Krishna, R. Screening metal–organic frameworks for mixture separations in fixed-bed adsorbers using a combined selectivity/capacity metric. *RSC Adv.* **7**, 35724-35737 (2017).
11. Krishna, R. Methodologies for screening and selection of crystalline microporous materials in mixture separations. *Sep. Purif. Technol.* **194**, 281-300 (2018).
12. Spek, A. Single-crystal structure validation with the program PLATON. *J. Appl. Crystallogr.* **36**, 7-13 (2003).
13. Xing, G., Liu, Q., Zhang, Y., Zhang, S. & Dong, Y. Microporous Zinc (II) Metal-Organic Framework with 6-Connected pcu Topology: Synthesis, Structure, and Gas Adsorption Properties. *Z. Anorg. Allg. Chem.* **641**, 1556-1559 (2015).
14. Wang, G. D. et al. One-step C2H4 Purification from Ternary C2H6/C2H4/C2H2 Mixtures by a Robust Metal-Organic Framework with Customized Pore Environment. *Angew. Chem. Int. Ed.* **61**, e202205427 (2022).
15. Ding, Q. et al. One-step Ethylene Purification from Ternary Mixtures in a Metal-Organic Framework with Customized Pore Chemistry and Shape. *Angew. Chem. Int. Ed.* **61**, e202208134 (2022).
16. Hao, H. G. et al. Simultaneous Trapping of C2H2 and C2H6 from a Ternary Mixture of C2H2/C2H4/C2H6 in a Robust Metal-Organic Framework for the Purification of C2H4. *Angew. Chem. Int. Ed.* **130**, 16299 –16303 (2018).
17. Xu, Z. et al. A robust Th-azole framework for highly efficient purification of C2H4 from a C2H4/C2H2/C2H6 mixture. *Nat. Commun.* **11**, 3163 (2020).
18. Zhu, B. et al. Pore Engineering for One-Step Ethylene Purification from a Three-Component Hydrocarbon Mixture. *J. Am. Chem. Soc.* **143**, 1485-1492 (2021).
19. Wang, Y. et al. One-step Ethylene Purification from an Acetylene/Ethylene/Ethane Ternary Mixture by Cyclopentadiene Cobalt-Functionalized Metal-Organic Frameworks. *Angew. Chem. Int. Ed.* **60**, 11350-11358 (2021).
20. Gu, X. W. et al. Immobilization of lewis basic sites into a stable ethane-selective MOF enabling one-step separation of ethylene from a ternary mixture. *J. Am. Chem. Soc.* **144**, 2614-2623 (2022).
21. Zhang, T. et al. General pore features for one-step C2H4 production from a C2 hydrocarbon mixture. *Chem. Commun.* **58**, 4954-4957 (2022).
22. Zhang, P. et al. Synergistic binding sites in a hybrid ultramicroporous material for one-step ethylene purification from ternary C(2) hydrocarbon mixtures. *Sci. Adv.* **8**, eabn9231 (2022).
23. Fan, L. et al. Rational Construction and Performance Regulation of an In(III)–Tetraisophthalate Framework for One-Step Adsorption-Phase Purification of C2H4 from C2 Hydrocarbons. *Inorg. Chem.* **60**, 10819-10829 (2021).
24. Jiang, Z. et al. An aromatic-rich cage-based MOF with inorganic chloride ions decorating the pore surface displaying the preferential adsorption of C2H2 and C2H6 over C2H4. *Inorg. Chem. Front.* **8**, 1243-1252 (2021).
25. Yang, S. Q. et al. Efficient Purification of Ethylene from C2 Hydrocarbons with an C2H6/C2H2-Selective Metal-Organic Framework. *ACS Appl. Mater. Interfaces* **13**, 962-969 (2021).
26. Liu, P. et al. Construction of saturated coordination titanium-based metal–organic framework for one-step C2H2/C2H6/C2H4 separation. *Sep. Purif. Technol.* **276**, 119284 (2021).

27. Xiong, Y. Y. et al. Dynamic Spacer Installation of Multifunctionalities into Metal–Organic Frameworks for Spontaneous One-Step Ethylene Purification from a Ternary C2-Hydrocarbons Mixture. *CCS Chem.* 023.202302698 (2023).

28. Wang, Y. T. et al. Guest-molecule-induced self-adaptive pore engineering facilitates purification of ethylene from ternary mixture. *Chem* 8, 3263–3274 (2022).

29. Ding, Q. et al. One-step ethylene purification from ternary mixture by synergetic molecular shape and size matching in a honeycomb-like ultramicroporous material. *Chem. Eng. J.* **450**, 138272 (2022).

30. Liu, J., Wang, H. & Li, J. Pillar-layer Zn-triazolate-dicarboxylate frameworks with a customized pore structure for efficient ethylene purification from ethylene/ethane/acetylene ternary mixtures. *Chem. Sci.* **14**, 5912-5917 (2023).

32. Cao, J. W. et al. One-step ethylene production from a four-component gas mixture by a single physisorbent. *Nat. Commun.* **12**, 6507 (2021).

33. Laha, S. et al. Tailoring Robust Al-MOF for Trapping C2H6 and C2H2 towards Efficient C2H4 Purification from Quaternary Mixtures. *Chem. Sci.* **13**, 7172-7180 (2022).
